# A Comparative Study of Survival Rate in High Grade Glioma Tumors Being Treated by Radiotherapy Alone Versus Chemoradiation With Nitrosourea

**DOI:** 10.5539/gjhs.v7n6p33

**Published:** 2015-03-25

**Authors:** Mohammad Houshyari, Farzaneh Hajalikhani, Afshin Rakhsha, Parastoo Hajian

**Affiliations:** 1 Department of Radiation oncology, Shahid Beheshti University of Medical Sciences, Tehran, Iran; 2 Department of Technology of Radiotherapy, School of Paramedical, Shahid Beheshti University of Medical Sciences, Tehran, Iran

**Keywords:** chemoradiotherapy, malignant glioma, radiotherapy, survival

## Abstract

**Background:**

In adults, malignant glioma (high-grade glioma) is one of the most common brain tumors. In spite of different types of treatment, the outcome is still not likely to be favorable. The aim of this study was to determine the difference between survival rate in adult patients with high grade glioma treated by radiotherapy only and those treated by a combination of radiotherapy and nitrosurea-based chemotherapy.

**Methods:**

This study was conducted using the records of 48 patients with grade 3 or 4 of glial brain tumor referred to the radiation-oncology ward of Shohada-e-Tajrish Hospital in Tehran, Iran from 2005 to 2012. The patients had undergone radiotherapy alone or adjuvant chemoradiation with nitrosourea. The median survival of patients after receiving the different types of treatment were evaluated using the Kaplan –Meier method and the log –rank exam. Data were analyzed using univariate analysis for median survival regarding to the patients’ age, gender, extent of surgery, Karnofsky performance status (KPS) with the Kaplan-Meier method, and the log-rank exam. We used the Cox-model for multivariate analysis.

**Results:**

Records of 48 patients were studied (34 men and 14 women). The mean survival were 18 months for men and 15.2 months for women (P = 0.05). Around 58% (28 patients) were more than 50 years old, and 42% (20 patients) were less than 50, and mean survival for the two age groups were 13 and 20 months, respectively (P < 0.001). Then, the patients were divided into three groups according to the extent of surgery, i.e., excisional biopsy (11 patients), stereotactic biopsy (22 patients), and resection (15 patients), and the mean survival for the three groups were 14.7, 17.3, and 18.8 months, respectively. There was no significant statistical difference for mean survival between the three groups (P = 0.23). The KPS was greater than 70% in 23 patients and less than 70% in 21 patients, and the mean survival for the former and latter groups were 17.6 and 16 months, respectively (P = 0.67), four patients had unknown KPS. Twenty patients received only radiotherapy, and chemoradiation was done for 28 patients, and the mean survival for the former and latter patients were 14.5 and 19 months, respectively (P = 0.15).

**Conclusion:**

In this study, we concluded that age was the only effective factor in the survival of the patients and that chemotherapy had no significant effect on the survival of the patients.

## 1. Introduction

Malignant glioma (high-grade glioma) is one of the most malignant tumors in adults (Goodenberger & Jenkins, 2012). The patients’ outcome is unlikely to be good, and the average duration of survival is less than 12 months. The most important prognostic factors for these tumors are age, Karnofsky performance status (KPS), mental status, and extent of surgery (Perez & Brady, 2013). For high grade tumors, maximal safe surgical resection followed by chemotherapy concurrent with radiotherapy is standard of care (Gondi, Vogelbaum, Grimm & Mehta, 2013), but there is still a challenge in the treatment of these tumors. Studies of the cells of glioblastoma suggest that there are some kinds of cells, such as stem cells, that are resistant to therapy, however, these cells are sensitive to temozolamide, carmustine (BCNU), and lomustine (CCNU) (Mihaliak et al., 2010). Although many improvements have been developed through drug studies, several drug agents are not able to penetrate into the brain`s blood flow and cerebro-spinal fluid (CSF) because of the blood brain barrier (Muldoon, Soussain, & Jahnke, 2007). Therefore, the effectiveness of chemotherapy may be decreased. However, utilizing specific doses of radiation (about 20-40 GY) to the brain can increase the density and penetration of chemotherapeutic agents into the blood brain barrier (Dexing, Guangfei, Hao, Yongwen & Gongli, 2001). Currently, chemoradiation is standard of care in treatment for patients with glioblastoma multiforme (GBM) (Gondi et al., 2013). One of the treatment options is the use of nitrosurea-based adjuvant chemotherapy after radiotherapy. The nitrosurea family is counted as an active agent against high-grade brain tumors. One of them is bischloroethyl nitrosourea (BCNU), and the other one is cyclohexyl chloroethyl nitrosourea (CCNU). Their biochemical structures are different (Tew, 2011). The aim of this study was to determine the difference between survival rate in adult patients with high grade glioma treated by radiotherapy only and those treated by a combination of radiotherapy and nitrosourea-based chemotherapy.

## 2. Materials and Methods

### 2.1 Research Design and Setting

This was a descriptive-analytic study. The records of the patients who were referred to the radiation-oncology ward of Shohada-e-Tajrish Hospital in Tehran, Iran from 2005 to 2012 were investigated carefully, and those with high grade glial brain tumors were selected.

The required sample size for this research was determined to be 94. The following formula was used to calculate the sample size:

n= Z^2^p(1-p)/d^2^,

where:

n= sample size

Z= normal distribution percentile

p= probability

d= confidence interval

### 2.2 Data Collection

The hospital records of the patients who had been referred to the radiation-oncology ward from 2005 to 2012 were carefully screened, and patients with high grade glial brain tumors were selected for further investigation. Their pathologies were grade 3 or 4 glial brain tumors, and the treatment protocol included radiotherapy alone or concurrent radiotherapy and chemotherapy with nitrosurea. Our pooled information consisted of age, gender, time of diagnosis, site of the tumor, extent of surgery, Karnofsky performance status (KPS), grade of tumor, histology, interval between beginning of symptoms to diagnosis, symptoms of disease (headache-vertigo-epilepsy-visual disturbance-upper and lower limb involvement), and the date of death. A total of 94 patients were included in the study, and then they were divided into group A and group B. Members of the A group were treated by radiotherapy only, and members of the B group were treated by adjuvant chemoradiation.

### 2.3 Ethical Consideration

A documented consent was prepared for every patient. Accordingly, the researchers were allowed to use the patients^`^ records, but the information was not disclosed to anyone. Ethical approval for the study was obtained from the ethical review group and the Deputy of Research at the Shahid Beheshti University of Medical Sciences under code 134.

### 2.4 Statistical Analyses

Data were analyzed using univariate analysis for mean survival with respect to the patient`s age, gender, extent of surgery, KPS with the Kaplan-Meier method, and log-rank exam. For multivariate analysis, we used the Cox-model to assess the effects of all variables in four treatment arms. Using Scan field residue, the establishment of a proportion hazard hypothesis was evaluated. The Data were analyzed using SPSS software, version 18.

## 3. Results

As was mentioned, the study was started with 94 patients according to the sample size, but during follow- up, 46 patients were excluded from the study because they did not come back to the hospital for their next visits with their physicians and/ or we could not locate them due to changes in their addresses or contact numbers. Thus, the study was continued with 48 patients. Comparing the 48 patients who remained in the study (34 men and 14 women) showed that the mean survival were 18 and 15.2 months for men and women, respectively (P = 0.05). In addition, 28 patients were more than 50 years old, and 20 patients were less than 50, mean survival for the two groups were 13 and 20 months, respectively (P < 0.000). We divided the patients into three groups according to the extent of surgery, i.e., excisional biopsy (11 patients), stereotactic tumor biopsy (22 patients), and resection (15 patients); the mean survival for these three groups were 14.7, 17.3, and 18.8 months, respectively (P = 0.23).

The KPS of patients manifested more than 70% in 23 patients and less than 70% in 21 patients and 4 patients had unknown KPS, the mean survival for the first and second groups were 17.6 and 16 months, respectively; there was no significant difference between the KPS of these two groups (P = 0.67). Moreover, 20 patients were treated only with radiotherapy and 28 patients received adjuvant chemoradiation. In the group that received adjuvant chemoradiation, five patients were given the procarbazine-ccnu-vincristine (PCV) regimen, six patients received BCNU and 17 patients were treated with CCNU. The mean survival for the patients who received only radiotherapy was 14.5 months. The mean survival for the patients who were treated with adjuvant chemoradiation with PCV, BCNU, and CCNU were 22.4, 16, and 19 months respectively. The median survival was calculated as 11 months for radiotherapy only, and for chemoradiation with PCV, BCNU, and CCNU, it was 24.4, 14.5, and 19.4 months, respectively (P = 0.46). Finally, all patients were divided into two groups, i.e., group A that had only radiotherapy and group B that had adjuvant chemoradiation. The mean survival in group A was14.5 months, and it was 19 months for the patients in group B; the median survival in group A was 11 months, and it was 19.4 months in group B (P = 0.15) (Table 1). Using Scan field residue, establishment of a proportion hazard hypothesis was evaluated, and it was true for all of the variables in the Cox-model (Table 2) (Table 3).

**Table 1:** Test of equality of survival distributions for the different levels of variables

Variable	Chi-Square	df	p-value
**Sex**	3.761	1	.052
**Age**	14.302	1	.000
**Level of surgery**	2.867	2	.238
**KPS**	.783	2	.676
**Different levels of four groups**	2.551	3	.466
**Different levels of two groups**	2.029	1	.154

**Table 2:** Omnibus Tests of Model Coefficients^a,b^

-2 Log Likelihood	Overall (Score)			Change From Previous Step			Change From Previous Block		

	Chi-square	df	Sig	Chi-square	df	Sig	Chi-square	df	Sig
208.810	19.043	9	0.025	18.419	9	0.031	18.419	9	0.31

a. Beginning Block Number 0, initial Log Likelihood function: -2 Log Likelihood: 227.229;

b. Beginning Block Number 1. Method= Enter.

**Table 3:** Variables in the Equation

	B	SE	Wald	df	Sig.	Exp (B)	95.0% CI for Exp (B)	

							Lower	Upper
**Group**			.714	3	.870			
**Group (1)**	-.020	.821	.001	1	.980	.980	.196	4.898
**Group (2)**	-.419	.643	.424	1	.515	.658	.187	2.318
**Group (3)**	-.270	.444	.371	1	.542	.763	.320	1.822
**Gender**	-.323	.418	.596	1	.440	.724	.319	1.643
**Surgery**			2.987	2	.225			
**Surgery (1)**	1.044	.605	2.972	1	.085	2.839	.867	9.300
**Surgery (2)**	.462	.481	.923	1	.337	1.588	.618	4.077
**KPS**			.259	2	.879			
**KPS (1)**	.095	.399	.057	1	.812	1.100	.503	2.405
**KPS (2)**	.378	.762	.246	1	.620	1.459	.328	6.497
**Age**	1.423	.496	8.226	1	.004	4.150	1.569	10.975

## 4. Discussion

Our findings suggest that the survival differed among patients who were given chemoradiation with nitrosurea compared with patients who only had radiotherapy. In the present study, the age of the patient was a predictable variable for longer survival. Being younger than 50 increased the survival significantly compared to those over 50. We did not find any significant statistical difference between the two groups of the study. Likewise our findings, results of a study aimed to evaluate the role of temozolamide in survival of patients with astrocytoma (grade 3), showed that only the age of patients was a predictive variable on survival rate (P = 0.001) and the role of surgery did not improve the outcome. The median survival for patients who were treated by chemoradiation using temozolamide was only 15 months versus radiotherapy only (13months), that was not a significant difference (Combs et al., 2008). However, a study conducted in Germany produced promising results. The mean age of patients was 58, and median survival was 14.5 months. The effects of different variables on the survival were: age (P = 0.001), extent of the surgery (P = 0.01), and chemotherapy (P = 0.01). BCNU, PCV, and temozolamide were the most chemotherapeutic agents that were used, but temozolamide was the most common drug that had been used. In their study, the role of chemotherapy was significant in outcome of the patients (Wehming et al., 2012). Likewise, in a randomized controlled trial with 674 patients, the two year survival was increased up to10% by following the PCV regimen versus radiotherapy only. (Median survival were 10 months and 9.5 months, respectively) (Medical research council brain tumor working party: randomized trial, *J Clin Oncol*, 2001). Results from a meta-analysis using pooled data from 3000 patients showed that increased survival rate occurred among patients who had nitrosurea for treatment (P = 0.001) (Stewart, 2002). The results showed that age had a significant effect on survival rate, while no such finding was obtained for other factors, such as KPS, gender, and extent of the surgery. Likewise, Tzu-Ming Yang et al., (2008) concluded that for patients with GBM who received temozolamide and radiotherapy simultaneously, median survival was improved to 30 months vs. 17 months for radiotherapy only, and the role of temozolamide on survival was significant (Yang et al., 2008). In another study, Stephanie et al., (2008) found that either age or extent of surgery were predictive variables that showed significant effects on survival, but it was not confident for chemotherapy (Combs et al., 2008). In one study with 205 patients who had different treatments (surgery alone, radiotherapy alone, chemotherapy alone, and adjuvant chemotherapy after radiotherapy), the median survival was 12 months, and age, KPS before surgery, location of tumor, radical surgery, chemotherapy, and radiotherapy all had significant effects on survival for patients with GBM. Surgery had the second most effective role on survival so that for radical surgery, survival was16 months and for partial surgery, it was 8 months. Median survival for those who received radiotherapy was 15 months vs. 8 months for patients who did not have such treatments. Temozolamide was not used extensively due to high cost, and nitrosurea-based chemotherapy was used more. Median survival after treating with adjuvant chemotherapy was 18 months vs. 10 months for patients that did not have any chemotherapy (Ma et al., 2009). For patients with GBM, median survival after surgery alone were 14 weeks, for chemotherapy alone (BCNU), 19 weeks, for radiotherapy alone, 37 weeks, for chemotherapy (BCNU) and radiotherapy, 49 weeks, and for chemotherapy (CCNU) and radiotherapy, 43 weeks. The authors concluded that surgery alone and chemotherapy alone did not have dramatic effects on survival and that there was no difference between chemotherapy plus radiotherapy vs. radiotherapy alone. There was not any significant difference between CCNU and BCNU. For radiotherapy alone and chemotherapy plus radiotherapy, these conclusions were true, but survival curves in 12 months were the same, but during 18 months, these curves were 10% for the adjuvant chemotherapy group and 4% for radiotherapy alone group. Response to chemotherapy depends on the age of the patients and the grade of the tumors. Thus, for patients over 60, the effects were not significant (Lonardi, Tosoni, & Brandes, 2005).

## 5. Conclusion

The aim of this study was to determine the difference between survival rate in adult patients with malignant glioma (high-grade glioma) treated by radiotherapy only and those treated by a combination of radiotherapy and nitrosurea-based chemotherapy. The main conclusion drawn from the results of this study was that there were no statistical dramatic effect in the chemoradiation group, with survival time increased by only a few months compared to radiotherapy alone. Also, it was concluded that age had a significant effect on survival, but this was not true for the other factors (KPS, gender, and extend of surgery). The presence of different results for chemotherapy in different studies requires further work with high sample sizes and an assessment of the role of chemotherapy and responses and evaluation of the roles of age, location, and extent of the surgery together. Finally, with regard to the early recurrence of malignant glioma (approximately six months) and the inability to endure additional irradiation, nitrosurea could be chosen as an optional treatment for these patients.
